# Supplementary material for: Heterotic grouping of wheat hybrids based on general and specific combining ability from line × tester analysis
Source: PeerJ. 2024 Sep 25;12:e18136. doi: 10.7717/peerj.18136 (PMC11438435; doi:10.7717/peerj.18136)
Supplement: Supplemental Information 2 [file peerj-12-18136-s002.docx]

**Suppl. Table 1.** Line x tester variance analyses for examined traits

| SOV | DF | PH | | SL | | GNS | | GWS | | TGW | | HI | | GY | |
| --- | --- | --- | --- | --- | --- | --- | --- | --- | --- | --- | --- | --- | --- | --- | --- |
|  |  | F_1_ | F_2_ | F_1_ | F_2_ | F_1_ | F_2_ | F_1_ | F_2_ | F_1_ | F_2_ | F_1_ | F_2_ | F_1_ | F_2_ |
| Replication | 2 | 1.16^ns^ | 61.88^ns^ | 0.67^ns^ | 0.89^ns^ | 47.62^*^ | 3.71^ns^ | 22.31^*^ | 4364.52^ns^ | 1538.67^ns^ | 10.46^ns^ | 3.71^ns^ | 22.31^*^ | 4364.52^ns^ | 1538.67^ns^ |
| Genotypes | 50 | 200.79^**^ | 200.91^**^ | 3.98^**^ | 1.02^**^ | 228.05^**^ | 79.36^**^ | 10.67^**^ | 92152.83^**^ | 13171.90^**^ | 26.85^**^ | 79.36^**^ | 10.67^**^ | 92152.83^**^ | 13171.90^**^ |
| Parents | 14 | 358.11^**^ | 276.41^**^ | 8.70^**^ | 1.57^**^ | 185.27^**^ | 67.72^**^ | 6.483^ns^ | 88769.25^**^ | 10078.56^**^ | 31.08^**^ | 67.72^**^ | 6.48^ns^ | 88769.25^**^ | 10078.56^**^ |
| Interaction | 1 | 608.97^**^ | 119.09^ns^ | 0.00^ns^ | 0.22^ns^ | 68.40^*^ | 538.54^**^ | 8.584^ns^ | 79241.27^**^ | 64122.51^**^ | 522.33^**^ | 538.54^**^ | 8.58^ns^ | 79241.27^**^ | 64122.51^**^ |
| Crosses | 35 | 126.19^**^ | 173.04^**^ | 2.20^**^ | 0.82^**^ | 249.73^**^ | 70.89^**^ | 12.41^**^ | 93875.16^**^ | 12953.51^**^ | 11.00^**^ | 70.89^**^ | 12.41^**^ | 93875.16^**^ | 12953.51^**^ |
| Lines | 11 | 276.25^**^ | 420.90^**^ | 2.17^ns^ | 1.05^ns^ | 548.70^**^ | 79.84^ns^ | 16.78^*^ | 116331.48^ns^ | 14536.24^ns^ | 18.04^ns^ | 79.84^ns^ | 16.78^*^ | 116331.48^ns^ | 14536.24^ns^ |
| Testers | 2 | 301.86^**^ | 53.29^ns^ | 12.73^**^ | 1.14^ns^ | 177.66^ns^ | 37.81^ns^ | 51.22^**^ | 103127.12^ns^ | 8573.62^ns^ | 3.06^ns^ | 37.81^ns^ | 51.22^**^ | 103127.12^ns^ | 8573.62^ns^ |
| Line x Tester | 22 | 35.20^**^ | 59.99^*^ | 1.26^**^ | 0.68^**^ | 106.80^**^ | 69.43^**^ | 6.70^ns^ | 81805.92^**^ | 12560.32^**^ | 8.20^ns^ | 69.43^**^ | 6.70^ns^ | 81805.92^**^ | 12560.32^**^ |
| Error | 100 | 10.34 | 31.96 | 0.47 | 0.31 | 12.24 | 4.43 | 6.03 | 2355.40 | 1314.16 | 5.06 | 4.43 | 6.03 | 2355.40 | 1314.16 |

^*^P<0,05 , ^**^ P < 0,01 (SOV: Source of variation; DF: Degree of freedom; PH: Plant height; SL: Spike length; GNS: Grain number per spike; GWS: Grain weight per spike; TGW: Thousand grain weight; HI: Harvest index; GY: Grain yield)
