# Supplementary material for: Heterotic grouping of wheat hybrids based on general and specific combining ability from line × tester analysis
Source: PeerJ. 2024 Sep 25;12:e18136. doi: 10.7717/peerj.18136 (PMC11438435; doi:10.7717/peerj.18136)
Supplement: Supplemental Information 3 [file peerj-12-18136-s003.docx]

**Suppl. Table 2.** Mean performance of parents for yield-related characteristics

| **Parents** | **PH** | | **SL** | | **GNS** | | **GWS** | | **TGW** | | **HI** | | **GY** | |
| --- | --- | --- | --- | --- | --- | --- | --- | --- | --- | --- | --- | --- | --- | --- |
|  | **F_1_** | **F_2_** | **F_1_** | **F_2_** | **F_1_** | **F_2_** | **F_1_** | **F_2_** | **F_1_** | **F_2_** | **F_1_** | **F_2_** | **F_1_** | **F_2_** |
| NZFE-64 | 83.5 | 88.8 | 12.5 | 11.2 | 60.5 | 60.7 | 1.72 | 2.52 | 28.74 | 42.45 | 34.40 | 50.15 | 717 | 672 |
| NZFE-63 | 77.5 | 91.9 | 17.0 | 10.9 | 58.9 | 66.3 | 1.37 | 2.56 | 29.83 | 35.84 | 26.62 | 49.44 | 264 | 580 |
| NZFE-62 | 84.2 | 101.6 | 12.9 | 11.2 | 66.9 | 57.6 | 2.31 | 2.50 | 29.79 | 39.70 | 42.28 | 47.54 | 607 | 578 |
| 4162-28 | 55.6 | 70.1 | 11.3 | 10.6 | 37.1 | 66.0 | 1.17 | 2.67 | 32.84 | 39.48 | 30.57 | 52.51 | 283 | 566 |
| 4166-1 | 64.1 | 71.6 | 10.7 | 9.7 | 42.6 | 56.9 | 1.37 | 1.98 | 28.15 | 41.18 | 32.81 | 49.33 | 378 | 538 |
| 4164-36 | 54.5 | 71.4 | 10.6 | 10.7 | 53.2 | 64.0 | 1.78 | 2.64 | 31.58 | 39.43 | 34.59 | 52.33 | 261 | 602 |
| NZFE-25 | 79.8 | 92.1 | 11.8 | 12.0 | 52.3 | 63.1 | 1.66 | 2.70 | 34.65 | 43.11 | 35.47 | 49.56 | 453 | 690 |
| NZFE-38 | 77.9 | 92.4 | 12.1 | 10.9 | 52.9 | 53.9 | 2.25 | 2.58 | 34.46 | 42.92 | 39.37 | 49.39 | 809 | 622 |
| NZFE-55 | 81.3 | 88.6 | 12.2 | 11.2 | 58.2 | 62.5 | 1.82 | 2.52 | 30.60 | 38.11 | 41.01 | 48.02 | 426 | 646 |
| NZFMT-14 | 79.0 | 95.6 | 11.1 | 11.6 | 54.6 | 64.8 | 1.51 | 2.81 | 26.47 | 42.98 | 37.36 | 50.06 | 628 | 648 |
| NZFMT-15 | 85.5 | 94.5 | 10.7 | 10.8 | 51.3 | 61.2 | 1.63 | 2.93 | 29.65 | 45.96 | 34.24 | 51.04 | 449 | 520 |
| NZFMT-21 | 87.9 | 93.0 | 11.9 | 11.4 | 50.6 | 66.3 | 1.34 | 2.99 | 31.44 | 48.25 | 26.18 | 50.50 | 404 | 616 |
| Tekirdağ | 80.2 | 84.7 | 11.4 | 10.8 | 67.1 | 65.6 | 2.10 | 3.13 | 32.83 | 42.54 | 38.88 | 52.33 | 712 | 560 |
| Renan | 91.7 | 95.1 | 12.2 | 12.5 | 54.3 | 65.6 | 1.85 | 3.03 | 33.02 | 43.54 | 35.60 | 49.27 | 561 | 650 |
| Esperia | 78.5 | 87.1 | 9.1 | 9.8 | 56.9 | 69.6 | 1.96 | 3.08 | 35.33 | 45.54 | 38.70 | 50.45 | 467 | 489 |

(PH: Plant height; SL: Spike length; GNS: Grain number per spike; GWS: Grain weight per spike; TGW: Thousand grain weight; HI: Harvest index; GY: Grain yield)
