# Supplementary material for: Heterotic grouping of wheat hybrids based on general and specific combining ability from line × tester analysis
Source: PeerJ. 2024 Sep 25;12:e18136. doi: 10.7717/peerj.18136 (PMC11438435; doi:10.7717/peerj.18136)
Supplement: Supplemental Information 4 [file peerj-12-18136-s004.docx]

**Suppl. Table 3.** General combining ability effects of parents for yield-related characteristics

| **Parents** | **PH** | | **SL** | | **GNS** | | **GWS** | | **TGW** | | **HI** | | **GY** | |
| --- | --- | --- | --- | --- | --- | --- | --- | --- | --- | --- | --- | --- | --- | --- |
|  | **F_1_** | **F_2_** | **F_1_** | **F_2_** | **F_1_** | **F_2_** | **F_1_** | **F_2_** | **F_1_** | **F_2_** | **F_1_** | **F_2_** | **F_1_** | **F_2_** |
| NZFE-64 | -0.12 | -0.35 | -0.46 | -0.11 | -1.68 | -1.89 | -0.11 | 0.17 | -0.23 | 0.74 | 0.14 | -1.69^*^ | 123.79^**^ | -60.24^**^ |
| NZFE-63 | 2.80^*^ | -1.03 | 0.43 | 0.18 | 10.05^**^ | 5.12^*^ | 0.41^**^ | -0.09 | -1.33 | -0.35 | 3.38^**^ | 0.83 | 145.56^**^ | 24.20^*^ |
| NZFE-62 | 8.86^**^ | 6.43^**^ | 0.69^**^ | -0.12 | -0.57 | -1.02 | -0.23^**^ | -0.26^*^ | -0.86 | -4.06^**^ | 2.81^**^ | -1.85^*^ | 50.34^**^ | 32.54^**^ |
| 4162-28 | -5.34^**^ | -8.31^**^ | 0.32 | 0.38^*^ | -12.87^**^ | -3.57 | -0.37^**^ | -0.20 | 5.72^**^ | -0.04 | 0.93 | -0.23 | 71.01^**^ | -9.35 |
| 4166-1 | -6.88^**^ | -12.91^**^ | -0.10 | -0.85^**^ | -9.20^**^ | -2.62 | -0.35^**^ | -0.15 | 0.40 | 1.21 | -4.08^**^ | 2.17^**^ | -135.21^**^ | -45.02^**^ |
| 4164-36 | -10.52^**^ | -9.65^**^ | 0.02 | -0.14 | -9.98^**^ | -1.76 | -0.19^*^ | -0.16 | 6.79^**^ | 0.47 | -5.28^**^ | 1.88^*^ | -135.21^**^ | -21.68 |
| NZFE-25 | 0.35 | 6.04^**^ | -0.52^**^ | -0.09 | -1.63 | 0.27 | -0.15^*^ | 0.19 | -3.39^**^ | 0.68 | -1.25 | -1.66^*^ | -132.21^**^ | 35.65^**^ |
| NZFE-38 | 3.21^**^ | 7.02^**^ | -0.64^**^ | 0.42^*^ | 2.90^**^ | 4.34^*^ | 0.24^**^ | 0.27^**^ | 0.34 | 0.30 | 1.68^*^ | 0.22 | 25.79 | -40.02^**^ |
| NZFE-55 | -1.04 | 6.58^**^ | -0.66^**^ | -0.11 | -0.76 | -1.34 | -0.38^**^ | -0.09 | -3.45^**^ | -0.99 | -3.81^**^ | -0.88 | -130.21^**^ | -20.57 |
| NZFMT-14 | 2.76^*^ | 1.28 | 0.21 | 0.37^*^ | 7.45^**^ | 3.55 | 0.17^*^ | 0.27^**^ | -1.19 | 0.90 | 1.72^*^ | 1.23 | 28.23 | 73.43^**^ |
| NZFMT-15 | 6.70^**^ | 1.94 | 0.71^**^ | 0.03 | 11.01^**^ | -1.05 | 0.52^**^ | -0.01 | -1.44 | 0.29 | 3.28^**^ | -0.26 | 148.34^**^ | -3.68 |
| NZFMT-21 | -0.78 | 2.97 | -0.00 | 0.04 | 5.29^**^ | -0.02 | 0.43^**^ | 0.06 | -1.35 | 0.87 | 0.47 | 0.24 | -60.21^**^ | 34.76^**^ |
| TEKİRDAĞ | -0.20 | -1.39 | -0.08 | -0.16 | 2.30^**^ | -0.97 | 0.16^**^ | -0.00 | -0.24 | -0.01 | -0.79^*^ | 0.30 | -34.60^**^ | -0.27 |
| RENAN | 2.99^**^ | 0.87 | 0.63^**^ | 0.19^*^ | -0.17 | -2.91^**^ | 0.02 | -0.18^**^ | 2.06^**^ | 0.30 | 1.16^**^ | -1.31^**^ | 61.65^**^ | -15.30^*^ |
| ESPERİA | -2.79^**^ | 0.52 | -0.55^**^ | -0.04 | -2.13^**^ | 3.88^**^ | -0.18^**^ | 0.18^**^ | -1.82^**^ | -0.29 | -0.37 | 1.01^*^ | -27.05^**^ | 15.56^*^ |

^*^P<0,05 , ^**^ P < 0,01 (PH: Plant height; SL: Spike length; GNS: Grain number per spike; GWS: Grain weight per spike; TGW: Thousand grain weight; HI: Harvest index; GY: Grain yield)
