# Supplementary material for: Heterotic grouping of wheat hybrids based on general and specific combining ability from line × tester analysis
Source: PeerJ. 2024 Sep 25;12:e18136. doi: 10.7717/peerj.18136 (PMC11438435; doi:10.7717/peerj.18136)
Supplement: Supplemental Information 5 [file peerj-12-18136-s005.docx]

**Suppl. Table 4.** Mean performance of hybrids for yield-related characteristics

| **Hybrids** | **PH** | | **SL** | | **GNS** | | **GWS** | | **TGW** | | **HI** | | **GY** | |
| --- | --- | --- | --- | --- | --- | --- | --- | --- | --- | --- | --- | --- | --- | --- |
|  | **F_1_** | **F_2_** | **F_1_** | **F_2_** | **F_1_** | **F_2_** | **F_1_** | **F_2_** | **F_1_** | **F_2_** | **F_1_** | **F_2_** | **F_1_** | **F_2_** |
| NZFE-64/Tekirdağ | 77.7 | 94.1 | 11.8 | 10.3 | 59.5 | 52.5 | 2.24 | 3.14 | 38.45 | 46.43 | 43.98 | 46.81 | 646 | 540 |
| NZFE-64/Renan | 82.8 | 77.5 | 11.8 | 10.8 | 53.7 | 58.9 | 2.03 | 3.00 | 33.16 | 47.87 | 36.11 | 46.80 | 703 | 583 |
| NZFE-64/Esperia | 84.5 | 96.8 | 9.4 | 11.4 | 49.7 | 68.3 | 1.81 | 3.81 | 33.81 | 46.29 | 38.29 | 50.14 | 656 | 626 |
| NZFE-63/Tekirdağ | 88.8 | 89.1 | 11.6 | 10.4 | 69.7 | 59.0 | 2.92 | 2.87 | 32.45 | 46.43 | 40.69 | 49.19 | 564 | 680 |
| NZFE-63/Renan | 86.4 | 90.4 | 13.7 | 11.0 | 67.0 | 67.3 | 2.65 | 2.90 | 34.37 | 44.44 | 43.73 | 50.11 | 768 | 728 |
| NZFE-63/Esperia | 78.6 | 86.9 | 11.4 | 11.9 | 61.3 | 74.4 | 2.07 | 3.40 | 35.30 | 46.44 | 43.68 | 52.01 | 738 | 595 |
| NZFE-62/Tekirdağ | 88.4 | 94.4 | 13.0 | 10.4 | 57.9 | 58.7 | 2.20 | 2.78 | 36.95 | 37.43 | 43.54 | 48.27 | 601 | 688 |
| NZFE-62/Renan | 97.2 | 96.6 | 12.7 | 11.1 | 60.6 | 59.3 | 2.11 | 2.67 | 35.37 | 45.11 | 37.47 | 45.81 | 542 | 700 |
| NZFE-62/Esperia | 86.4 | 97.8 | 11.8 | 10.9 | 47.6 | 64.4 | 1.43 | 3.20 | 31.21 | 43.64 | 45.37 | 49.19 | 642 | 640 |
| 4162-28/Tekirdağ | 76.3 | 77.2 | 11.8 | 10.6 | 50.9 | 51.1 | 1.83 | 2.57 | 39.50 | 47.50 | 43.40 | 48.77 | 847 | 647 |
| 4162-28/Renan | 78.8 | 86.0 | 12.8 | 11.8 | 38.8 | 58.1 | 1.78 | 2.86 | 44.55 | 45.77 | 44.90 | 47.91 | 750 | 672 |
| 4162-28/Esperia | 74.3 | 81.3 | 11.9 | 11.6 | 39.6 | 65.5 | 1.71 | 3.39 | 39.23 | 44.98 | 32.46 | 51.46 | 250 | 583 |
| 4166-1/Tekirdağ | 77.4 | 73.1 | 11.9 | 10.5 | 44.5 | 61.7 | 1.93 | 3.09 | 37.25 | 47.64 | 32.30 | 50.80 | 311 | 502 |
| 4166-1/Renan | 81.6 | 84.9 | 13.4 | 10.4 | 51.8 | 55.7 | 1.91 | 2.90 | 38.61 | 48.64 | 37.69 | 51.96 | 596 | 623 |
| 4166-1/Esperia | 65.8 | 72.8 | 9.9 | 9.4 | 43.9 | 60.1 | 1.53 | 3.01 | 31.45 | 45.71 | 35.72 | 52.58 | 320 | 671 |
| 4164-36/Tekirdağ | 65.2 | 78.5 | 11.5 | 10.7 | 40.6 | 60.0 | 1.71 | 3.06 | 41.62 | 47.95 | 29.90 | 53.60 | 215 | 591 |
| 4164-36/Renan | 76.2 | 82.4 | 12.5 | 11.1 | 46.6 | 55.0 | 2.09 | 2.79 | 44.65 | 46.50 | 38.78 | 48.36 | 585 | 585 |
| 4164-36/Esperia | 72.4 | 79.7 | 11.5 | 10.6 | 50.7 | 65.1 | 2.05 | 3.12 | 40.23 | 45.32 | 33.45 | 52.50 | 429 | 689 |
| NZFE-25/Tekirdağ | 82.5 | 94.7 | 11.6 | 11.2 | 66.7 | 62.2 | 2.46 | 3.42 | 30.47 | 48.50 | 41.49 | 47.16 | 538 | 688 |
| NZFE-25/Renan | 84.3 | 97.4 | 11.4 | 10.8 | 50.3 | 57.7 | 2.06 | 3.06 | 36.31 | 45.55 | 42.45 | 45.85 | 490 | 654 |
| NZFE-25/Esperia | 79.7 | 95.4 | 10.8 | 10.5 | 46.0 | 66.3 | 1.44 | 3.54 | 29.15 | 46.38 | 30.29 | 50.84 | 209 | 696 |
| NZFE-38/Tekirdağ | 88.2 | 98.7 | 10.8 | 11.5 | 53.7 | 70.6 | 1.98 | 3.48 | 34.29 | 47.24 | 31.23 | 50.14 | 302 | 612 |
| NZFE-38/Renan | 84.5 | 94.5 | 12.3 | 11.4 | 61.2 | 61.5 | 2.69 | 3.41 | 39.32 | 47.10 | 46.54 | 50.44 | 796 | 519 |
| NZFE-38/Esperia | 82.3 | 97.3 | 10.4 | 11.2 | 61.6 | 66.4 | 2.47 | 3.37 | 33.55 | 44.92 | 45.25 | 48.89 | 613 | 680 |
| NZFE-55/Tekirdağ | 79.8 | 95.6 | 11.4 | 10.2 | 55.0 | 59.2 | 1.97 | 2.98 | 30.43 | 44.80 | 37.85 | 50.16 | 564 | 698 |
| NZFE-55/Renan | 82.0 | 95.4 | 11.4 | 11.7 | 54.9 | 59.9 | 1.41 | 2.92 | 36.30 | 44.68 | 34.63 | 46.65 | 366 | 471 |
| NZFE-55/Esperia | 80.5 | 98.3 | 10.8 | 10.6 | 55.7 | 62.3 | 1.91 | 3.26 | 29.04 | 45.92 | 34.06 | 49.36 | 313 | 699 |
| NZFMT-14/Tekirdağ | 85.5 | 86.5 | 11.9 | 11.7 | 68.9 | 68.1 | 2.40 | 3.76 | 31.25 | 46.71 | 36.02 | 52.58 | 368 | 721 |
| NZFMT-14/Renan | 87.9 | 91.5 | 12.8 | 11.1 | 60.8 | 60.6 | 2.36 | 3.15 | 37.83 | 47.98 | 43.03 | 49.92 | 631 | 719 |
| NZFMT-14/Esperia | 80.2 | 95.3 | 11.5 | 11.1 | 60.5 | 67.3 | 2.19 | 3.35 | 33.45 | 46.38 | 44.09 | 50.02 | 719 | 710 |
| NZFMT-15/Tekirdağ | 87.2 | 89.8 | 12.6 | 10.7 | 76.9 | 62.6 | 3.25 | 3.29 | 33.40 | 46.59 | 45.22 | 51.52 | 694 | 649 |
| NZFMT-15/Renan | 91.9 | 95.8 | 12.3 | 11.2 | 60.4 | 58.1 | 2.26 | 3.07 | 32.28 | 46.85 | 39.36 | 47.94 | 601 | 584 |
| NZFMT-15/Esperia | 86.4 | 89.7 | 12.7 | 10.9 | 63.6 | 61.6 | 2.47 | 3.04 | 36.12 | 45.79 | 43.24 | 48.59 | 784 | 686 |
| NZFMT-21/Tekirdağ | 82.1 | 89.6 | 10.8 | 11.2 | 54.8 | 64.3 | 2.73 | 3.27 | 35.57 | 46.16 | 36.79 | 49.86 | 469 | 703 |
| NZFMT-21/Renan | 83.9 | 95.9 | 12.4 | 11.1 | 63.4 | 54.7 | 2.54 | 2.90 | 36.45 | 46.55 | 41.07 | 47.77 | 447 | 699 |
| NZFMT-21/Esperia | 77.0 | 92.9 | 12.2 | 10.6 | 65.6 | 66.4 | 2.44 | 3.44 | 30.04 | 48.27 | 41.52 | 51.90 | 537 | 633 |

(PH: Plant height; SL: Spike length; GNS: Grain number per spike; GWS: Grain weight per spike; TGW: Thousand grain weight; HI: Harvest index; GY: Grain yield)
