# Supplementary material for: Heterotic grouping of wheat hybrids based on general and specific combining ability from line × tester analysis
Source: PeerJ. 2024 Sep 25;12:e18136. doi: 10.7717/peerj.18136 (PMC11438435; doi:10.7717/peerj.18136)
Supplement: Supplemental Information 6 [file peerj-12-18136-s006.docx]

**Suppl. Table 5.** Specific combining ability effects of hybrids for yield-related characteristics.

| **Hybrids** | **PH** | | **SL** | | **GNS** | | **GWS** | | **TGW** | | **HI** | | **GY** | |
| --- | --- | --- | --- | --- | --- | --- | --- | --- | --- | --- | --- | --- | --- | --- |
|  | **F_1_** | **F_2_** | **F_1_** | **F_2_** | **F_1_** | **F_2_** | **F_1_** | **F_2_** | **F_1_** | **F_2_** | **F_1_** | **F_2_** | **F_1_** | **F_2_** |
| NZFE-64/Tekirdağ | -3.77^*^ | 6.01 | 0.55 | -0.41 | 2.90 | -6.44 | 0.05 | -0.17 | 3.54^**^ | -0.42 | 5.31^**^ | -1.41 | 12.27 | -42.95^*^ |
| NZFE-64/Renan | -1.87 | -12.84^**^ | -0.23 | -0.23 | -0.43 | 1.89 | -0.01 | -0.14 | -4.04^**^ | 0.71 | -4.51^**^ | 0.19 | -27.31 | 15.41 |
| NZFE-64/Esperia | 5.64^**^ | 6.83^*^ | -0.32 | 0.64 | -2.47 | 4.54 | -0.04 | 0.31 | 0.50 | -0.28 | -0.80 | 1.21 | 15.05 | 27.55 |
| NZFE-63/Tekirdağ | 4.37^*^ | 1.69 | -0.54 | -0.57 | 1.37 | -6.92 | 0.21 | -0.18 | -1.36 | 0.67 | -1.22 | -1.54 | -91.18^**^ | 12.27 |
| NZFE-63/Renan | -1.16 | 0.73 | 0.84^*^ | -0.28 | 1.17 | 3.28 | 0.08 | 0.02 | -1.73 | -1.62 | -0.13 | 0.99 | 16.57 | 75.63^**^ |
| NZFE-63/Esperia | -3.21 | -2.42 | -0.31 | 0.85^*^ | -2.53 | 3.63 | -0.30^*^ | 0.16 | 3.08^*^ | 0.95 | 1.35 | 0.55 | 74.60^**^ | -87.90^**^ |
| NZFE-62/Tekirdağ | -2.08 | -0.50 | 0.61 | -0.27 | 0.25 | -1.15 | 0.12 | -0.10 | 2.67^*^ | -4.62^**^ | 2.20 | 0.21 | 41.05 | 12.27 |
| NZFE-62/Renan | 3.55 | -0.49 | -0.44 | 0.12 | 5.39^**^ | 1.45 | 0.18 | -0.03 | -1.20 | 2.76^*^ | -5.81^**^ | -0.63 | -114.87^**^ | 38.96 |
| NZFE-62/Esperia | -1.47 | 0.99 | -0.16 | 0.15 | -5.64^**^ | -0.30 | -0.31^*^ | 0.13 | -1.47 | 1.87 | 3.61^**^ | 0.42 | 73.82^**^ | -51.23^**^ |
| 4162-28/Tekirdağ | 0.02 | -2.93 | -0.26 | -0.53 | 5.49^**^ | -6.13 | -0.11 | -0.36^*^ | -1.35 | 1.43 | 3.94^**^ | -0.91 | 265.71^**^ | 13.49 |
| 4162-28/Renan | -0.65 | 3.64 | -0.01 | 0.25 | -4.14^*^ | 2.74 | -0.01 | 0.10 | 1.40 | -0.61 | 3.49^**^ | -0.16 | 72.80^*^ | 53.52^**^ |
| 4162-28/Esperia | 0.63 | -0.71 | 0.27 | 0.28 | -1.34 | 3.39 | 0.12 | 0.27 | -0.04 | -0.82 | -7.42^**^ | 1.07 | -338.51^**^ | -67.01^**^ |
| 4166-1/Tekirdağ | 2.69 | -2.43 | 0.26 | 0.57 | -4.52^*^ | 3.51 | -0.02 | 0.09 | 1.72 | 0.32 | -2.15 | -1.28 | -63.40^*^ | -96.51^**^ |
| 4166-1/Renan | 3.66 | 7.11^*^ | 1.01^*^ | 0.08 | 5.21^*^ | -0.58 | 0.11 | 0.08 | 0.78 | 1.02 | 1.30 | 1.49 | 125.35^**^ | 39.85 |
| 4166-1/Esperia | -6.36^**^ | -4.68 | -1.27^**^ | -0.65^*^ | -0.69 | -2.93 | -0.08 | -0.17 | -2.50 | -1.34 | 0.86 | -0.22 | -61.95^*^ | 56.66^**^ |
| 4164-36/Tekirdağ | -5.84^**^ | -0.32 | -0.23 | 0.06 | -7.67^**^ | 0.96 | -0.40^**^ | 0.07 | -0.31 | 1.37 | -3.35^**^ | 1.81 | -160.06^**^ | -30.18 |
| 4164-36/Renan | 1.96 | 1.32 | 0.06 | 0.11 | 0.83 | -2.17 | 0.12 | -0.02 | 0.42 | -0.39 | 3.58^**^ | -1.81 | 113.68^**^ | -21.48 |
| 4164-36/Esperia | 3.88^*^ | -1.00 | 0.17 | -0.16 | 6.83^**^ | 1.21 | 0.28^*^ | -0.05 | -0.11 | -0.98 | -0.22 | -0.00 | 46.38 | 51.66^*^ |
| NZFE-25/Tekirdağ | 0.56 | 0.26 | 0.42 | 0.51 | 10.08^**^ | 1.13 | 0.31^*^ | 0.09 | -1.27 | 1.70 | 4.20^**^ | -1.09 | 160.27^**^ | 8.82 |
| NZFE-25/Renan | -0.87 | 0.70 | -0.50 | -0.20 | -3.89 | -1.47 | 0.06 | -0.10 | 2.27 | -1.56 | 3.22^**^ | -0.78 | 15.68 | -9.81 |
| NZFE-25/Esperia | 0.31 | -0.96 | 0.08 | -0.31 | -6.19^**^ | 0.34 | -0.37^**^ | 0.02 | -1.01 | -0.14 | -7.41^**^ | 1.87 | -175.95^**^ | 0.99 |
| NZFE-38/Tekirdağ | 3.39 | 3.25 | -0.29 | 0.27 | -7.42^**^ | 5.39 | -0.56^**^ | 0.06 | -1.19 | 0.83 | -8.99^**^ | 0.02 | -234.06^**^ | 8.49 |
| NZFE-38/Renan | -3.50 | -3.21 | 0.52 | -0.12 | 2.55 | -1.74 | 0.29^*^ | 0.17 | 1.54 | 0.38 | 4.38^**^ | 1.93 | 164.02^**^ | -69.15^**^ |
| NZFE-38/Esperia | 0.11 | -0.03 | -0.23 | -0.15 | 4.88^*^ | -3.65 | 0.27^*^ | -0.23 | -0.34 | -1.21 | 4.61^**^ | -1.95 | 70.05^*^ | 60.66^**^ |
| NZFE-55/Tekirdağ | -0.72 | 0.58 | 0.28 | -0.48 | -2.52 | -0.33 | 0.05 | -0.07 | -1.26 | -0.32 | 3.13^*^ | 1.14 | 184.60^**^ | 75.38^**^ |
| NZFE-55/Renan | -1.78 | -1.91 | -0.43 | 0.64 | -0.15 | 2.34 | -0.37^**^ | 0.05 | 2.31 | -0.75 | -2.05 | -0.76 | -110.31^**^ | -136.26^**^ |
| NZFE-55/Esperia | 2.50 | 1.33 | 0.15 | -0.16 | 2.68 | -2.01 | 0.33^*^ | 0.02 | -1.06 | 1.07 | -1.08 | -0.38 | -74.29^**^ | 60.88^**^ |
| NZFMT-14/Tekirdağ | 1.15 | -3.22 | -0.08 | 0.54 | 3.17 | 3.75 | -0.08 | 0.34 | -2.69^*^ | -0.30 | -4.24^**^ | 1.44 | -170.18^**^ | 4.71 |
| NZFMT-14/Renan | 0.38 | -0.44 | 0.10 | -0.37 | -2.43 | -1.85 | 0.02 | -0.09 | 1.59 | 0.66 | 0.83 | 0.40 | -3.09 | 17.74 |
| NZFMT-14/Esperia | -1.53 | 3.67 | -0.02 | -0.17 | -0.73 | -1.90 | 0.05 | -0.25 | 1.10 | -0.36 | 3.41^**^ | -1.84 | 173.27^**^ | -22.45 |
| NZFMT-15/Tekirdağ | -1.09 | -0.58 | 0.18 | -0.08 | 7.63^**^ | 2.78 | 0.43^**^ | 0.16 | -0.30 | 0.19 | 3.40^**^ | 1.88 | 36.05 | 9.49 |
| NZFMT-15/Renan | 0.41 | 3.20 | -0.90^*^ | 0.07 | -6.40^**^ | 0.28 | -0.42^**^ | 0.12 | -3.71^**^ | 0.14 | -4.41^**^ | -0.10 | -153.87^**^ | -40.15 |
| NZFMT-15/Esperia | 0.69 | -2.62 | 0.71 | 0.00 | -1.23 | -3.07 | -0.01 | -0.28 | 4.01^**^ | -0.33 | 1.00 | -1.78 | 117.82^**^ | 30.66 |
| NZFMT-21/Tekirdağ | 1.33 | -1.81 | -0.90^*^ | 0.38 | -8.74^**^ | 3.45 | -0.00 | 0.07 | 1.79 | -0.82 | -2.22 | -0.28 | 18.93 | 24.71 |
| NZFMT-21/Renan | -0.14 | 2.20 | -0.02 | -0.07 | 2.29 | -4.18 | -0.05 | -0.13 | 0.37 | -0.74 | 0.12 | -0.76 | -98.65^**^ | 35.74 |
| NZFMT-21/Esperia | -1.19 | -0.39 | 0.93^*^ | -0.31 | 6.45^**^ | 0.73 | 0.05 | 0.06 | -2.16 | 1.57 | 2.10 | 1.04 | 79.71^**^ | -60.45^**^ |

^*^P<0,05 , ^**^ P < 0,01 (PH: Plant height; SL: Spike length; GNS: Grain number per spike; GWS: Grain weight per spike; TGW: Thousand grain weight; HI: Harvest index; GY: Grain yield)
