# Supplementary material for: Heterotic grouping of wheat hybrids based on general and specific combining ability from line × tester analysis
Source: PeerJ. 2024 Sep 25;12:e18136. doi: 10.7717/peerj.18136 (PMC11438435; doi:10.7717/peerj.18136)
Supplement: Supplemental Information 7 [file peerj-12-18136-s007.docx]

**Suppl. Table 6.** Heterosis values of hybrids for yield-related characteristics

| **Hybrids** | **PH** | | **SL** | | **GNS** | | **GWS** | | **TGW** | | **HI** | | **GY** | |
| --- | --- | --- | --- | --- | --- | --- | --- | --- | --- | --- | --- | --- | --- | --- |
|  | **F_1_** | **F_2_** | **F_1_** | **F_2_** | **F_1_** | **F_2_** | **F_1_** | **F_2_** | **F_1_** | **F_2_** | **F_1_** | **F_2_** | **F_1_** | **F_2_** |
| NZFE-64/Tekirdağ | -5.13 | 8.47^**^ | -1.67 | -6.36^*^ | -6.74 | -16.93^**^ | 17.40 | 11.15^*^ | 24.90^**^ | 9.25^**^ | 20.05^**^ | -8.65^**^ | -9.59 | -12.34^**^ |
| NZFE-64/Renan | -5.48^*^ | -15.72^**^ | -4.84 | -9.24^**^ | -6.45 | -6.80^*^ | 13.86 | 8.11^**^ | 7.38 | 11.33^**^ | 3.18 | -5.86^**^ | 10.02 | -11.80^**^ |
| NZFE-64/Esperia | 4.32 | 10.06^**^ | -12.96^**^ | 8.57^**^ | -15.33^*^ | 4.75 | -1.52 | 35.90^**^ | 5.54 | 5.20^*^ | 4.76 | -0.31 | 10.81 | 7.75 |
| NZFE-63/Tekirdağ | 12.55^**^ | 0.91 | -18.31^**^ | -4.59 | 10.63 | -10.61^**^ | 68.47^**^ | 0.95 | 3.57 | 18.47^**^ | 24.25^**^ | -3.32^**^ | 15.57 | 19.30^**^ |
| NZFE-63/Renan | 2.13 | -3.32 | -6.16^*^ | -5.98^*^ | 18.37^**^ | 1.97 | 64.60^**^ | 3.65 | 9.37 | 11.97^**^ | 40.56^**^ | 1.53^**^ | 86.18^**^ | 18.37^**^ |
| NZFE-63/Esperia | 0.77 | -2.91 | -12.98^**^ | 14.42^**^ | 5.87 | 9.41^**^ | 24.40^*^ | 20.39^**^ | 8.35 | 14.13^**^ | 33.74^**^ | 4.13^**^ | 101.92^**^ | 11.21^*^ |
| NZFE-62/Tekirdağ | 7.54^**^ | 1.34 | 6.56 | -5.45^*^ | -13.58^*^ | -4.71 | -0.32 | -1.14 | 18.01^**^ | -8.97^**^ | 7.31 | -3.33^**^ | -8.87 | 20.91^**^ |
| NZFE-62/Renan | 10.45^**^ | -1.78 | 0.79 | -6.72^**^ | 0.00 | -3.73 | 1.59 | -3.37 | 12.63^*^ | 8.39^**^ | -3.77 | -5.35^**^ | -7.19 | 14.01^**^ |
| NZFE-62/Esperia | 6.14^*^ | 3.66 | 7.27 | 3.81 | -23.10^**^ | 1.26 | -33.15^**^ | 14.51^**^ | -4.15 | 2.39 | 12.05^**^ | 0.40 | 19.55 | 19.85^**^ |
| 4162-28/Tekirdağ | 12.37^**^ | -0.26 | 3.51 | -0.93 | -2.30 | -22.34^**^ | 11.68 | -11.11^**^ | 20.30^**^ | 15.83^**^ | 24.98^**^ | -6.96^**^ | 70.25^**^ | 14.92^**^ |
| 4162-28/Renan | 6.92^*^ | 4.12 | 8.47^*^ | 1.72 | -15.10 | -11.70^**^ | 18.01 | 0.49 | 35.29^**^ | 10.26^**^ | 35.71^**^ | -5.85^**^ | 77.73^**^ | 10.53^*^ |
| 4162-28/Esperia | 10.73^**^ | 3.44 | 16.67^**^ | 13.73^**^ | -15.74^*^ | -3.39 | 9.53 | 17.87^**^ | 15.09^*^ | 5.81^**^ | -6.29 | -0.03 | -33.33 | 10.42^*^ |
| 4166-1/Tekirdağ | 7.20^*^ | -6.46 | 7.21 | 1.94 | -18.94^**^ | 0.65 | 11.43 | 20.95^**^ | 22.17^**^ | 13.81^**^ | -9.90^*^ | -0.06 | -42.94^**^ | -8.56 |
| 4166-1/Renan | 4.75 | 1.86 | 16.52^**^ | -6.31^*^ | 6.80 | -9.14^**^ | 19.10 | 15.85^**^ | 26.24^**^ | 14.83^**^ | 10.20^*^ | 5.40^**^ | 26.94^*^ | 4.88 |
| 4166-1/Esperia | -7.71^*^ | -8.25^*^ | 0.00 | -4.08 | -11.85 | -5.06 | -8.12 | 18.87^**^ | -0.91 | 5.42^*^ | -0.08 | 5.39^**^ | -24.26 | 30.54^**^ |
| 4164-36/Tekirdağ | -3.26 | 0.58 | 4.55 | -0.93 | -32.56^**^ | -7.41^**^ | -11.70 | 6.06^*^ | 29.23^**^ | 16.98^**^ | -18.60^**^ | 2.43^**^ | -55.81^**^ | 1.72 |
| 4164-36/Renan | 4.24 | -1.02 | 9.65^**^ | -4.31 | -13.38^*^ | -15.12^**^ | 15.16 | -1.59 | 38.24^**^ | 12.08^**^ | 10.51^*^ | -4.80^**^ | 42.34^**^ | -6.55 |
| 4164-36/Esperia | 8.87^**^ | 0.57 | 16.16^**^ | 2.91 | -7.99 | -2.54 | 9.63 | 8.83^**^ | 20.25^**^ | 6.66^**^ | -8.72 | 2.17^**^ | 17.86 | 26.19^**^ |
| NZFE-25/Tekirdağ | 3.13 | 7.13^*^ | 0.00 | -1.75 | 11.73^*^ | -3.42 | 30.71^**^ | 17.47^**^ | -9.69 | 13.24^**^ | 11.60^*^ | -7.43^**^ | -7.64 | 10.08^*^ |
| NZFE-25/Renan | -1.75 | 4.06 | -5.00 | -12.20^**^ | -5.63 | -10.40^**^ | 17.67 | 6.78^*^ | 7.31 | 5.12^*^ | 19.47^**^ | -7.21^**^ | -3.35 | -2.39 |
| NZFE-25/Esperia | 0.63 | 6.47^*^ | 2.86 | -3.67 | -15.75^*^ | -0.15 | -20.24^*^ | 22.35^**^ | -16.69^**^ | 4.62^*^ | -18.32^**^ | 1.67^**^ | -54.57^**^ | 17.97^**^ |
| NZFE-38/Tekirdağ | 11.50^**^ | 11.46^**^ | -8.47^*^ | 5.50^*^ | -10.50 | 18.06^**^ | -8.78 | 21.83^**^ | 1.92 | 10.55^**^ | -20.18^**^ | -1.40^**^ | -60.29^**^ | 3.55 |
| NZFE-38/Renan | -0.35 | 0.80 | 0.82 | -2.56 | 14.18^*^ | 2.84 | 31.14^**^ | 21.68^**^ | 16.54^**^ | 8.95^**^ | 24.16^**^ | 2.24^**^ | 16.20 | -18.40^**^ |
| NZFE-38/Esperia | 5.24 | 8.41^**^ | -1.89 | 7.69^**^ | 12.20 | 7.44^**^ | 17.59^*^ | 19.13^**^ | -3.85 | 1.56 | 15.91^**^ | -2.06^**^ | -3.92 | 22.30^**^ |
| NZFE-55/Tekirdağ | -1.24 | 10.33^**^ | -3.39 | -7.27^**^ | -12.28^*^ | -7.64^**^ | 0.66 | 5.38 | -4.05 | 11.08^**^ | -5.23 | -0.03 | -0.88 | 15.75^**^ |
| NZFE-55/Renan | -5.20^*^ | 3.86 | -6.56 | -1.68 | -2.49 | -6.55^*^ | -23.13^*^ | 5.41 | 14.12^*^ | 9.43^**^ | -9.60^*^ | -4.10^**^ | -25.84^*^ | -27.31^**^ |
| NZFE-55/Esperia | 0.75 | 11.90^**^ | 0.93 | 0.95 | -3.30 | -5.75^*^ | 1.17 | 16.20^**^ | -11.91^*^ | 9.78^**^ | -14.54^**^ | 0.24 | -29.90^*^ | 23.06^**^ |
| NZFMT-14/Tekirdağ | 7.41^**^ | -4.05 | 5.31 | 4.46 | 13.14^*^ | 4.45 | 32.85^**^ | 26.63^**^ | 5.40 | 9.24^**^ | -5.53 | 2.71^**^ | -45.07^**^ | 19.37^**^ |
| NZFMT-14/Renan | 2.93 | -4.04 | 9.40^**^ | -8.26^**^ | 11.56 | -7.06^**^ | 40.32^**^ | 7.84^**^ | 27.18^**^ | 10.91^**^ | 17.96^**^ | 0.52 | 6.14 | 10.79^**^ |
| NZFMT-14/Esperia | 1.78 | 4.32 | 13.86^**^ | 3.74 | 8.42 | 0.15 | 26.18^*^ | 13.73^**^ | 8.25 | 4.79^*^ | 15.93^**^ | -0.47 | 31.32^**^ | 24.78^**^ |
| NZFMT-15/Tekirdağ | 5.19^*^ | 0.22 | 13.51^**^ | -0.93 | 29.90^**^ | -1.26 | 74.53^**^ | 8.51^**^ | 6.91 | 5.29^*^ | 23.69^**^ | -0.31 | 19.55 | 20.19^**^ |
| NZFMT-15/Renan | 3.72 | 1.05 | 6.96 | -4.27 | 14.39^*^ | -8.36^**^ | 29.96^**^ | 3.09 | 3.02 | 4.69^*^ | 12.71^*^ | -4.42^**^ | 19.01 | -0.17 |
| NZFMT-15/Esperia | 5.37^*^ | -1.21 | 28.28^**^ | 5.83^*^ | 17.56^**^ | -5.81^*^ | 37.79^**^ | 0.93 | 11.17 | 0.09 | 18.57^**^ | -4.25^**^ | 71.18^**^ | 35.84^**^ |
| NZFMT-21/Tekirdağ | -2.38 | 0.84 | -7.69^*^ | 0.90 | -6.96 | -2.58 | 58.70^**^ | 6.80^*^ | 10.69 | 1.67 | 13.10^*^ | -3.01^**^ | -15.95 | 19.56^**^ |
| NZFMT-21/Renan | -6.57^**^ | 1.97 | 2.48 | -7.50^**^ | 20.76^**^ | -17.12^**^ | 59.22^**^ | -3.69 | 13.09^*^ | 1.42 | 32.96^**^ | -4.25^**^ | -7.36 | 10.43^*^ |
| NZFMT-21/Esperia | -7.45^**^ | 3.16 | 16.19^**^ | 0.00 | 21.93^**^ | -2.35 | 48.06^**^ | 13.30^**^ | -10.02 | 2.92 | 27.99^**^ | 2.83^**^ | 23.31 | 14.47^**^ |

^*^P<0,05 , ^**^ P < 0,01 (PH: Plant height; SL: Spike length; GNS: Grain number per spike; GWS: Grain weight per spike; TGW: Thousand grain weight; HI: Harvest index; GY: Grain yield)

Table 7. Heterobeltiosis values of hybrids for yield-related characteristics

| **Hybrids** | **PH** | | **SL** | | **GNS** | | **GWS** | | **TGW** | | **HI** | | **GY** | |
| --- | --- | --- | --- | --- | --- | --- | --- | --- | --- | --- | --- | --- | --- | --- |
|  | **F_1_** | **F_2_** | **F_1_** | **F_2_** | **F_1_** | **F_2_** | **F_1_** | **F_2_** | **F_1_** | **F_2_** | **F_1_** | **F_2_** | **F_1_** | **F_2_** |
| NZFE-64/Tekirdağ | -6.95^*^ | 5.97 | -5.60 | -8.04^**^ | -11.33 | -19.97^**^ | 6.77 | 0.35 | 17.12^*^ | 9.14^**^ | 13.12^*^ | -10.55^**^ | -9.90 | -19.64^**^ |
| NZFE-64/Renan | -9.71^**^ | -18.51^**^ | -5.60 | -13.60^**^ | -11.24 | -10.21^**^ | 9.85 | -0.96 | 0.42 | 9.94^**^ | 1.44 | -6.69^**^ | -1.95 | -13.24^**^ |
| NZFE-64/Esperia | 1.20 | 9.01^*^ | -24.80^**^ | 1.79 | -17.85^**^ | -1.87 | -7.52 | 23.44^**^ | -4.30 | 1.65 | -1.06 | -0.61 | -8.51 | -6.85 |
| NZFE-63/Tekirdağ | 10.72^**^ | -3.05 | -31.76^**^ | -4.59 | 3.87 | -10.06^**^ | 39.32^**^ | -8.15^**^ | -1.16 | 9.14^**^ | 4.66 | -5.99^**^ | -20.79^*^ | 17.24^**^ |
| NZFE-63/Renan | -5.78^*^ | -4.94 | -19.41^**^ | -12.00^**^ | 13.75^*^ | 2.59 | 43.48^**^ | -4.26 | 4.09 | 2.07 | 22.84^**^ | 1.36^**^ | 36.90^**^ | 12.00^**^ |
| NZFE-63/Esperia | 0.13 | -5.44 | -32.94^**^ | 9.17^**^ | 4.07 | 6.90^*^ | 5.83^*^ | 10.24^**^ | -0.08 | 1.98 | 12.87^*^ | 3.09^**^ | 58.03^**^ | 2.59 |
| NZFE-62/Tekirdağ | 4.99 | -7.09^*^ | 0.78 | -7.14^*^ | -13.71^*^ | -11.46^**^ | -4.89 | -11.09^**^ | 12.55 | -12.01^**^ | 3.00 | -7.76^**^ | -15.59 | 19.03^**^ |
| NZFE-62/Renan | 6.00^*^ | -4.92 | -1.55 | -11.20^**^ | -9.42 | -10.56^**^ | -8.57 | -11.79^**^ | 7.12 | 3.61 | -11.37^*^ | -7.01^**^ | -10.71 | 7.69 |
| NZFE-62/Esperia | 2.61 | -3.74 | -8.53^*^ | -2.68 | -28.85^**^ | -7.47^**^ | -38.27^**^ | 3.63 | -11.66 | -4.17 | 7.31 | -2.50^**^ | 5.77 | 10.73^*^ |
| 4162-28/Tekirdağ | -4.86 | -8.85^*^ | 3.51 | -1.85 | -24.14^**^ | -22.10^**^ | -12.96 | -17.68^**^ | 20.28^**^ | 11.66^**^ | 11.63^*^ | -7.11^**^ | 18.96 | 14.31^**^ |
| 4162-28/Renan | -14.07^**^ | -9.57^**^ | 4.92 | -5.60^*^ | -28.55^**^ | -11.43^**^ | -3.52 | -5.48 | 34.92^**^ | 5.12^*^ | 26.13^**^ | -8.75^**^ | 33.69^*^ | 3.38 |
| 4162-28/Esperia | -5.35 | -6.66 | 5.31 | 9.43^**^ | -30.40^**^ | -5.89^*^ | -12.42 | 9.89^**^ | 11.04 | -1.23 | -16.12^**^ | -1.99^**^ | -46.47^**^ | 3.00 |
| 4166-1/Tekirdağ | -3.49 | -13.70^**^ | 4.39 | -2.78 | -33.68^**^ | -6.52^*^ | -7.96 | -1.25 | 13.46 | 11.99^**^ | -16.94^**^ | -2.92^**^ | -56.32^**^ | -10.36^*^ |
| 4166-1/Renan | -11.01^**^ | -10.73^**^ | 9.84^*^ | -16.80^**^ | -4.60 | -15.61^**^ | 3.63 | -4.16 | 16.93^*^ | 11.71^**^ | 5.88 | 5.33^**^ | 6.24 | -4.15 |
| 4166-1/Esperia | -16.18^**^ | -16.42^**^ | -7.48 | -4.08 | -22.85^**^ | -13.65^**^ | -21.93 | -2.40 | -10.98 | 0.37 | -7.68 | 4.22^**^ | -31.48^*^ | 24.72^**^ |
| 4164-36/Tekirdağ | -18.70^**^ | -7.32^*^ | 0.88 | -0.93 | -39.49^**^ | -8.54^**^ | -18.35 | -2.14 | 26.77^**^ | 12.72^**^ | -23.09^**^ | 2.43^**^ | -69.80^**^ | -1.83 |
| 4164-36/Renan | -16.90^**^ | -13.35^**^ | 2.46 | -11.20^**^ | -14.18 | -16.16^**^ | 13.10 | -7.80^*^ | 35.22^**^ | 6.80^**^ | 8.94 | -7.58^**^ | 4.28 | -10.00^*^ |
| 4164-36/Esperia | -7.77^*^ | -8.50^*^ | 8.49 | -0.93 | -10.90 | -6.47^*^ | 4.75 | 1.07 | 13.87^*^ | -0.48 | -13.56^**^ | 0.33 | -8.14 | 14.45^**^ |
| NZFE-25/Tekirdağ | 2.87 | 2.82 | -1.69 | -6.67^*^ | -0.60 | -5.18 | 17.06^**^ | 9.40^**^ | -12.06 | 12.50^**^ | 6.70 | -9.88^**^ | -24.44^*^ | -0.29 |
| NZFE-25/Renan | -8.07^**^ | 2.42 | -6.56 | -13.60^**^ | -7.37 | -12.04^**^ | 11.75 | 0.96 | 4.79 | 4.62 | 19.24^**^ | -7.48^**^ | -12.66 | -5.22 |
| NZFE-25/Esperia | -0.13 | 3.58 | -8.47^*^ | -12.50^**^ | -19.16^**^ | -4.74 | -26.28^*^ | 14.65^**^ | -17.49^**^ | 1.84 | -21.72^**^ | 0.78^*^ | -55.25^**^ | 0.87 |
| NZFE-38/Tekirdağ | 9.98^**^ | 6.82^*^ | -10.74^**^ | 5.50 | -19.97^**^ | 7.62^*^ | -11.86 | 11.16^**^ | -0.49 | 10.07^**^ | -20.68^**^ | -4.17^**^ | -62.67^**^ | -1.61 |
| NZFE-38/Renan | -7.85^**^ | -0.63 | 0.82 | -8.80^**^ | 12.71 | -6.25^*^ | 19.37^**^ | 12.72^**^ | 14.10^*^ | 8.18^**^ | 18.21^**^ | 2.12^**^ | -1.61 | -20.15^**^ |
| NZFE-38/Esperia | 4.84 | 5.30 | -14.05^**^ | 2.75 | 8.26 | -4.60 | 9.91^*^ | 9.40^**^ | -5.04 | -1.36 | 14.92^**^ | -3.09^**^ | -24.23^**^ | 9.32^*^ |
| NZFE-55/Tekirdağ | -1.85 | 7.90^*^ | -6.56 | -8.93^**^ | -18.03^**^ | -9.76^**^ | -6.05 | -4.86 | -7.31 | 5.31^*^ | -7.69 | -4.15^**^ | -20.79^*^ | 8.05 |
| NZFE-55/Renan | -10.58^**^ | 0.32 | -6.56 | -6.40^*^ | -5.67 | -8.69^**^ | -23.71^*^ | -3.44 | 9.93 | 2.62 | -15.56^**^ | -5.31^**^ | -34.76^**^ | -27.54^**^ |
| NZFE-55/Esperia | -0.98 | 10.95^**^ | -11.48^**^ | -5.36 | -4.30 | -10.49^**^ | -2.40 | 5.54 | -17.80^**^ | 0.83 | -16.95^**^ | -2.16^**^ | -32.98^*^ | 8.20 |
| NZFMT-14/Tekirdağ | 6.61^*^ | -9.52^**^ | 4.39 | 0.86 | 2.68 | 3.81 | 14.30^**^ | 20.24^**^ | -4.81 | 8.68^**^ | -7.37 | 0.49 | -48.31^**^ | 11.27^*^ |
| NZFMT-14/Renan | -4.14 | -4.29 | 4.92 | -11.20^**^ | 11.36 | -7.62^*^ | 27.56^**^ | 4.03 | 14.57^*^ | 10.20^**^ | 15.17^**^ | -0.28 | 0.48 | 10.62^*^ |
| NZFMT-14/Esperia | 1.52 | -0.31 | 3.60 | -4.31 | 6.33 | -3.30 | 11.86^*^ | 8.72^**^ | -5.32 | 1.84 | 13.93^**^ | -0.85^**^ | 14.49 | 9.57^*^ |
| NZFMT-15/Tekirdağ | 1.99 | -4.97 | 10.53^*^ | -0.93 | 14.61 | -4.57 | 55.15^**^ | 5.18 | 1.74 | 1.37 | 16.30^**^ | -1.53^**^ | -2.53 | 15.89^**^ |
| NZFMT-15/Renan | 0.22 | 0.74 | 0.82 | -10.40^**^ | 11.23 | -11.43^**^ | 22.36^**^ | 1.52 | -2.24 | 1.94 | 10.56 | -6.08^**^ | 7.13 | -10.15^*^ |
| NZFMT-15/Esperia | 1.05 | -5.08 | 18.69^**^ | 0.93 | 11.78 | -11.49^**^ | 26.38^**^ | -1.52 | 2.24 | -0.37 | 11.74^*^ | -4.80^**^ | 67.88^**^ | 31.92^**^ |
| NZFMT-21/Tekirdağ | -6.60^*^ | -3.66 | -9.24^*^ | -1.75 | -18.33^**^ | -3.02 | 30.03^**^ | 4.48 | 8.35 | -4.33 | -5.38 | -4.71^**^ | -34.13^**^ | 14.12^**^ |
| NZFMT-21/Renan | -8.51^**^ | 0.84 | 1.64 | -11.20^**^ | 16.76^*^ | -17.50^**^ | 37.41^**^ | -4.26 | 10.39 | -3.52 | 15.37^**^ | -5.41^**^ | -20.32 | 7.54 |
| NZFMT-21/Esperia | -12.40^**^ | -0.11 | 2.52 | -7.02^*^ | 15.29^*^ | -4.60 | 24.74^**^ | 11.57^**^ | -14.97^*^ | 0.04 | 7.29 | 2.78^**^ | 14.99 | 2.76 |

Table 8. Inbreeding depression values of hybrids for yield-related characteristics

| **Hybrids** | **PH** | **SL** | **GNS** | **GWS** | **TGW** | **HI** | **GY** |
| --- | --- | --- | --- | --- | --- | --- | --- |
| NZFE-64/Tekirdağ | -21.11^**^ | 12.71^**^ | 11.76^**^ | -40.13^**^ | -20.75^**^ | -6.42^*^ | 16.41^**^ |
| NZFE-64/Renan | 6.40^*^ | 8.47^**^ | -9.68^*^ | -47.76^**^ | -44.36^**^ | -29.59^**^ | 17.07^**^ |
| NZFE-64/Esperia | -14.56^**^ | -21.28^**^ | -37.42^**^ | -110.50^**^ | -36.91^**^ | -30.96^**^ | 4.57 |
| NZFE-63/Tekirdağ | -0.34 | 10.34^**^ | 15.35^**^ | 1.71 | -43.08^**^ | -20.89^**^ | -20.57^**^ |
| NZFE-63/Renan | -4.63 | 19.71^**^ | -0.45 | -9.36 | -29.30^**^ | -14.60^**^ | 5.21^*^ |
| NZFE-63/Esperia | -10.56^**^ | -4.39 | -21.37^**^ | -64.30^**^ | -31.56^**^ | -19.07^**^ | 19.38^**^ |
| NZFE-62/Tekirdağ | -6.79^**^ | 20.00^**^ | -1.38 | -26.58^**^ | -1.30 | -10.85^**^ | -14.48^**^ |
| NZFE-62/Renan | 0.62 | 12.60^**^ | 2.15 | -26.42^**^ | -27.54^**^ | -22.26^**^ | -29.15^**^ |
| NZFE-62/Esperia | -13.19^**^ | 7.63^**^ | -35.29^**^ | -124.19^**^ | -39.83^**^ | -8.42^**^ | 0.31 |
| 4162-28/Tekirdağ | -1.18 | 10.17^**^ | -0.39 | -41.02^**^ | -20.25^**^ | -12.36^**^ | 23.61^**^ |
| 4162-28/Renan | -9.14^**^ | 7.81^**^ | -49.74^**^ | -60.55^**^ | -2.74 | -6.70^**^ | 10.40^**^ |
| 4162-28/Esperia | -9.42^**^ | 2.52 | -65.40^**^ | -97.90^**^ | -14.66^**^ | -58.55^**^ | -133.20^**^ |
| 4166-1/Tekirdağ | 5.56^*^ | 11.76^**^ | -38.65^**^ | -59.97^**^ | -27.89^**^ | -57.29^**^ | -61.41^**^ |
| 4166-1/Renan | -4.04 | 22.39^**^ | -7.53 | -51.57^**^ | -25.98^**^ | -37.85^**^ | -4.53 |
| 4166-1/Esperia | -10.64^**^ | 5.05 | -36.90^**^ | -97.18^**^ | -45.34^**^ | -47.18^**^ | -109.69^**^ |
| 4164-36/Tekirdağ | -20.40^**^ | 6.96^**^ | -47.78^**^ | -78.69^**^ | -15.21^**^ | -79.24^**^ | -174.88^**^ |
| 4164-36/Renan | -8.14^**^ | 11.20^**^ | -18.03^**^ | -33.60^**^ | -4.14 | -24.70^**^ | 0.00 |
| 4164-36/Esperia | -10.08^**^ | 7.83^**^ | -28.40^**^ | -52.17^**^ | -12.65^**^ | -56.96^**^ | -60.61^**^ |
| NZFE-25/Tekirdağ | -14.79^**^ | 3.45 | 6.75 | -39.33^**^ | -59.17^**^ | -13.67^**^ | -27.88^**^ |
| NZFE-25/Renan | -15.54^**^ | 5.26^*^ | -14.71^**^ | -48.06^**^ | -25.45^**^ | -8.01^**^ | -33.47^**^ |
| NZFE-25/Esperia | -19.70^**^ | 2.78 | -44.13^**^ | -145.28^**^ | -59.11^**^ | -67.84^**^ | -233.01^**^ |
| NZFE-38/Tekirdağ | -11.90^**^ | -6.48^*^ | -31.47^**^ | -75.25^**^ | -37.77^**^ | -60.56^**^ | -102.65^**^ |
| NZFE-38/Renan | -11.83^**^ | 7.32^**^ | -0.49 | -26.98^**^ | -19.79^**^ | -8.37^**^ | 34.80^**^ |
| NZFE-38/Esperia | -18.23^**^ | -7.69^**^ | -7.79^*^ | -36.42^**^ | -33.89^**^ | -8.05^**^ | -10.93^**^ |
| NZFE-55/Tekirdağ | -19.80^**^ | 10.53^**^ | -7.64 | -50.99^**^ | -47.22^**^ | -32.50^**^ | -23.76^**^ |
| NZFE-55/Renan | -16.34^**^ | -2.63 | -9.11^*^ | -107.45^**^ | -23.09^**^ | -34.73^**^ | -28.69^**^ |
| NZFE-55/Esperia | -22.11^**^ | 1.85 | -11.85^**^ | -70.56^**^ | -58.13^**^ | -44.92^**^ | -123.32^**^ |
| NZFMT-14/Tekirdağ | -1.17 | 1.68 | 1.16 | -56.84^**^ | -49.47^**^ | -45.99^**^ | -95.92^**^ |
| NZFMT-14/Renan | -4.10 | 13.28^**^ | 0.33 | -33.66^**^ | -26.83^**^ | -16.01^**^ | -13.95^**^ |
| NZFMT-14/Esperia | -18.83^**^ | 3.48 | -11.24^**^ | -53.29^**^ | -38.65^**^ | -13.45^**^ | 1.25 |
| NZFMT-15/Tekirdağ | -2.98 | 15.08^**^ | 18.60^**^ | -1.08 | -39.49^**^ | -13.94^**^ | 6.48^*^ |
| NZFMT-15/Renan | -4.24 | 8.94^**^ | 3.81 | -35.97^**^ | -45.14^**^ | -21.80^**^ | 2.83 |
| NZFMT-15/Esperia | -3.82 | 14.17^**^ | 3.14 | -22.90^**^ | -26.77^**^ | -12.37^**^ | 12.50^**^ |
| NZFMT-21/Tekirdağ | -9.14^**^ | -3.70 | -17.34^**^ | -19.79^**^ | -29.77^**^ | -35.54^**^ | -49.89^**^ |
| NZFMT-21/Renan | -14.30^**^ | 10.48^**^ | 13.72^**^ | -14.18^**^ | -27.71^**^ | -16.31^**^ | -56.38^**^ |
| NZFMT-21/Esperia | -20.65^**^ | 13.11^**^ | -1.22 | -41.07^**^ | -60.69^**^ | -25.01^**^ | -17.88^**^ |
