# Supplementary material for: Heterotic grouping of wheat hybrids based on general and specific combining ability from line × tester analysis
Source: PeerJ. 2024 Sep 25;12:e18136. doi: 10.7717/peerj.18136 (PMC11438435; doi:10.7717/peerj.18136)
Supplement: Supplemental Information 9 [file peerj-12-18136-s009.docx]

**Suppl. Table 8.** Inbreeding depression values of hybrids for yield-related characteristics

| **Hybrids** | **PH** | **SL** | **GNS** | **GWS** | **TGW** | **HI** | **GY** |
| --- | --- | --- | --- | --- | --- | --- | --- |
| NZFE-64/Tekirdağ | -21.11^**^ | 12.71^**^ | 11.76^**^ | -40.13^**^ | -20.75^**^ | -6.42^*^ | 16.41^**^ |
| NZFE-64/Renan | 6.40^*^ | 8.47^**^ | -9.68^*^ | -47.76^**^ | -44.36^**^ | -29.59^**^ | 17.07^**^ |
| NZFE-64/Esperia | -14.56^**^ | -21.28^**^ | -37.42^**^ | -110.50^**^ | -36.91^**^ | -30.96^**^ | 4.57 |
| NZFE-63/Tekirdağ | -0.34 | 10.34^**^ | 15.35^**^ | 1.71 | -43.08^**^ | -20.89^**^ | -20.57^**^ |
| NZFE-63/Renan | -4.63 | 19.71^**^ | -0.45 | -9.36 | -29.30^**^ | -14.60^**^ | 5.21^*^ |
| NZFE-63/Esperia | -10.56^**^ | -4.39 | -21.37^**^ | -64.30^**^ | -31.56^**^ | -19.07^**^ | 19.38^**^ |
| NZFE-62/Tekirdağ | -6.79^**^ | 20.00^**^ | -1.38 | -26.58^**^ | -1.30 | -10.85^**^ | -14.48^**^ |
| NZFE-62/Renan | 0.62 | 12.60^**^ | 2.15 | -26.42^**^ | -27.54^**^ | -22.26^**^ | -29.15^**^ |
| NZFE-62/Esperia | -13.19^**^ | 7.63^**^ | -35.29^**^ | -124.19^**^ | -39.83^**^ | -8.42^**^ | 0.31 |
| 4162-28/Tekirdağ | -1.18 | 10.17^**^ | -0.39 | -41.02^**^ | -20.25^**^ | -12.36^**^ | 23.61^**^ |
| 4162-28/Renan | -9.14^**^ | 7.81^**^ | -49.74^**^ | -60.55^**^ | -2.74 | -6.70^**^ | 10.40^**^ |
| 4162-28/Esperia | -9.42^**^ | 2.52 | -65.40^**^ | -97.90^**^ | -14.66^**^ | -58.55^**^ | -133.20^**^ |
| 4166-1/Tekirdağ | 5.56^*^ | 11.76^**^ | -38.65^**^ | -59.97^**^ | -27.89^**^ | -57.29^**^ | -61.41^**^ |
| 4166-1/Renan | -4.04 | 22.39^**^ | -7.53 | -51.57^**^ | -25.98^**^ | -37.85^**^ | -4.53 |
| 4166-1/Esperia | -10.64^**^ | 5.05 | -36.90^**^ | -97.18^**^ | -45.34^**^ | -47.18^**^ | -109.69^**^ |
| 4164-36/Tekirdağ | -20.40^**^ | 6.96^**^ | -47.78^**^ | -78.69^**^ | -15.21^**^ | -79.24^**^ | -174.88^**^ |
| 4164-36/Renan | -8.14^**^ | 11.20^**^ | -18.03^**^ | -33.60^**^ | -4.14 | -24.70^**^ | 0.00 |
| 4164-36/Esperia | -10.08^**^ | 7.83^**^ | -28.40^**^ | -52.17^**^ | -12.65^**^ | -56.96^**^ | -60.61^**^ |
| NZFE-25/Tekirdağ | -14.79^**^ | 3.45 | 6.75 | -39.33^**^ | -59.17^**^ | -13.67^**^ | -27.88^**^ |
| NZFE-25/Renan | -15.54^**^ | 5.26^*^ | -14.71^**^ | -48.06^**^ | -25.45^**^ | -8.01^**^ | -33.47^**^ |
| NZFE-25/Esperia | -19.70^**^ | 2.78 | -44.13^**^ | -145.28^**^ | -59.11^**^ | -67.84^**^ | -233.01^**^ |
| NZFE-38/Tekirdağ | -11.90^**^ | -6.48^*^ | -31.47^**^ | -75.25^**^ | -37.77^**^ | -60.56^**^ | -102.65^**^ |
| NZFE-38/Renan | -11.83^**^ | 7.32^**^ | -0.49 | -26.98^**^ | -19.79^**^ | -8.37^**^ | 34.80^**^ |
| NZFE-38/Esperia | -18.23^**^ | -7.69^**^ | -7.79^*^ | -36.42^**^ | -33.89^**^ | -8.05^**^ | -10.93^**^ |
| NZFE-55/Tekirdağ | -19.80^**^ | 10.53^**^ | -7.64 | -50.99^**^ | -47.22^**^ | -32.50^**^ | -23.76^**^ |
| NZFE-55/Renan | -16.34^**^ | -2.63 | -9.11^*^ | -107.45^**^ | -23.09^**^ | -34.73^**^ | -28.69^**^ |
| NZFE-55/Esperia | -22.11^**^ | 1.85 | -11.85^**^ | -70.56^**^ | -58.13^**^ | -44.92^**^ | -123.32^**^ |
| NZFMT-14/Tekirdağ | -1.17 | 1.68 | 1.16 | -56.84^**^ | -49.47^**^ | -45.99^**^ | -95.92^**^ |
| NZFMT-14/Renan | -4.10 | 13.28^**^ | 0.33 | -33.66^**^ | -26.83^**^ | -16.01^**^ | -13.95^**^ |
| NZFMT-14/Esperia | -18.83^**^ | 3.48 | -11.24^**^ | -53.29^**^ | -38.65^**^ | -13.45^**^ | 1.25 |
| NZFMT-15/Tekirdağ | -2.98 | 15.08^**^ | 18.60^**^ | -1.08 | -39.49^**^ | -13.94^**^ | 6.48^*^ |
| NZFMT-15/Renan | -4.24 | 8.94^**^ | 3.81 | -35.97^**^ | -45.14^**^ | -21.80^**^ | 2.83 |
| NZFMT-15/Esperia | -3.82 | 14.17^**^ | 3.14 | -22.90^**^ | -26.77^**^ | -12.37^**^ | 12.50^**^ |
| NZFMT-21/Tekirdağ | -9.14^**^ | -3.70 | -17.34^**^ | -19.79^**^ | -29.77^**^ | -35.54^**^ | -49.89^**^ |
| NZFMT-21/Renan | -14.30^**^ | 10.48^**^ | 13.72^**^ | -14.18^**^ | -27.71^**^ | -16.31^**^ | -56.38^**^ |
| NZFMT-21/Esperia | -20.65^**^ | 13.11^**^ | -1.22 | -41.07^**^ | -60.69^**^ | -25.01^**^ | -17.88^**^ |

^*^P<0,05 , ^**^ P < 0,01 (PH: Plant height; SL: Spike length; GNS: Grain number per spike; GWS: Grain weight per spike; TGW: Thousand grain weight; HI: Harvest index; GY: Grain yield)
